# Selective Glycopolymer Inhibitors of Galectin-3: Supportive Anti-Cancer Agents Protecting Monocytes and Preserving Interferon-Gamma Function

**DOI:** 10.2147/IJN.S503381

**Published:** 2025-05-24

**Authors:** Marcela Filipová, Marina Rodrigues Tavares, Michaela Hovorková, Viktoria Heine, Pavlína Nekvasilová, Vladimír Křen, Tomáš Etrych, Petr Chytil, Pavla Bojarová

**Affiliations:** 1 Department of Biological Models, Institute of Macromolecular Chemistry of the Czech Academy of Sciences, Prague, Czech Republic; 2 Department of Biomedical Polymers, Institute of Macromolecular Chemistry of the Czech Academy of Sciences, Prague, Czech Republic; 3 Laboratory of Biotransformation, Institute of Microbiology of the Czech Academy of Sciences, Prague, Czech Republic; 4 Department of Genetics and Microbiology, Faculty of Science, Charles University, Prague, Czech Republic; 5 Department of Health Care Disciplines and Population Protection, Faculty of Biomedical Engineering, Czech Technical University in Prague, Kladno, Czech Republic

**Keywords:** carbohydrate, galectin-3, glycopolymer, interferon-gamma, monocyte, tumor microenvironment

## Abstract

**Introduction:**

The immunosuppressive roles of galectin-3 (Gal-3) in carcinogenesis make this lectin an attractive target for pharmacological inhibition in immunotherapy. Although current clinical immunotherapies appear promising in the treatment of solid tumors, their efficacy is significantly weakened by the hostile immunosuppressive tumor microenvironment (TME). Gal-3, a prominent TME modulator, efficiently subverts the elimination of cancer, either directly by inducing apoptosis of immune cells or indirectly by binding essential effector molecules, such as interferon-gamma (IFNγ).

**Methods:**

*N*-(2-Hydroxypropyl)methacrylamide (HPMA)-based glycopolymers bearing poly-*N*-acetyllactosamine-derived tetrasaccharide ligands of Gal-3 were designed, synthesized, and characterized using high-performance liquid chromatography, dynamic light scattering, UV-Vis spectrophotometry, gel permeation chromatography, nuclear magnetic resonance, high-resolution mass spectrometry and CCK-8 assay for evaluation of glycopolymer non-toxicity. Pro-immunogenic effects of purified glycopolymers were tested by apoptotic assay using flow cytometry, competitive ELISA, and in vitro cell-free INFγ-based assay.

**Results:**

All tested glycopolymers completely inhibited Gal-3-induced apoptosis of monocytes/macrophages, of which the M1 subtype is responsible for eliminating cancer cells during immunotherapy. Moreover, the glycopolymers suppressed Gal-3-induced capture of glycosylated IFNγ by competitive inhibition to Gal-3 carbohydrate recognition domain (CRD), which enables further inherent biological activities of this effector, such as differentiation of monocytes into M1 macrophages and repolarization of M2-macrophages to the M1 state.

**Conclusion:**

The prepared glycopolymers are promising inhibitors of Gal-3 and may serve as important supportive anti-cancer nanosystems enabling the infiltration of proinflammatory macrophages and the reprogramming of unwanted M2 macrophages into the M1 subtype.

## Graphical Abstract

**Figure:**
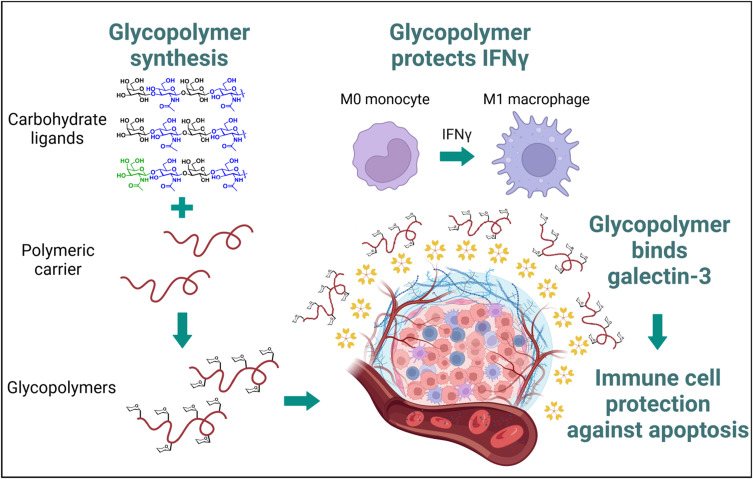

## Introduction

The efficacy of current clinical cancer immunotherapies is severely limited by the immunosuppressive tumor microenvironment (TME), in which tumor monocytes play an important role. As the most common type of immune cells in solid tumors,^1^ monocytes are subjected to intense investigation due to their precise role in tumorigenesis. The molecularly distinct immunostimulatory (M1) and immunosuppressive (M2) subpopulations of monocytes have been found in human lung adenocarcinomas and in some colorectal tumors. In particular, the immunostimulatory monocytes (M1) are associated with the ability to reverse the resistance to anti-PD-1 treatment in mouse models and to increase the clinical response to anti-PD-1 immunotherapy.^2^

TME itself preferentially influences the polarization and differentiation of monocytes towards M2 (anti-inflammatory) macrophages. It has been shown that the M1/M2 phenotype of macrophages is already molecularly determined at the monocyte stage.^2^ This M1/M2 presetting is strongly influenced by the cocktail of molecules present in the TME. For example, chemokines such as IL-4 and IL-13 drive the polarization and differentiation of monocytes into the M2 phenotype of macrophages,^3^ whereas IL-6, IL-12 or IFNγ drive macrophage polarization into the M1 (pro-inflammatory) phenotype.^3–5^ Therefore, modulation of TME to contain pro-inflammatory molecules is urgently needed.

Galectins (Gal-) belong to the group of TME proteins whose influence on tumorigenesis has been intensively studied. These carbohydrate-binding proteins contain a conserved C-terminal carbohydrate recognition domain (CRD), which recognizes β-galactoside-capped ligands, and they are highly expressed in most cancers.^6^ Due to their ability to form lattices, they can promote cooperativity and molecular regulation on the cell surface. Currently, the most studied galectins are Gal-1 and Gal-3, which play important roles in cancer biology and tumorigenesis, such as promotion of tumor growth, angiogenesis, metastatic spread, and inhibiting cancer immunity.^6^,^7^

Gal-1 belongs to the immunosuppressive molecules of TME, binding to glycosylated receptors of immune cells, thereby weakening immune cell function.^8^ It also stimulates the expression of programmed cell death ligand-1 (PD-L1),^9^ whose constitutive expression is responsible for the resistance to checkpoint inhibition in cancer immunotherapy.^10^ Similarly, Gal-3 was shown to block macrophage infiltration into the tumor mass and to act as a shield against immune cell infiltration into the tumor; inside these tumors, the M2 phenotype of macrophages was predominant.^11^ Although the M2 phenotype of macrophages could potentially be reprogrammed to M1 by IFNγ,^3^ Gal-3 may efficiently abrogate this process by binding glycosylated IFNγ through the Gal-3 carbohydrate recognition domain (CRD).^12^ Therefore, Gal-3-bound IFNγ is not available in TME, thus preventing the reprogramming of the M2/M1 phenotype of macrophages, and also the activation (priming) of macrophages to secrete increased levels of proinflammatory cytokines and decreased amounts of anti-inflammatory cytokines to enhance antitumor activity.^13^ Therefore, effective inhibition of Gal-3 may support macrophage infiltration into the tumor mass, as well as possible reprogramming of pro-tumor M2 macrophages towards anti-tumor M1 macrophages by IFNγ. Moreover, IFNγ available in TME directly mediates CD8-T cell cytotoxic functions and motility^14^ Recently, blocking of Gal-3 with a protein-based small-molecule inhibitor was shown to prevent diminished binding of anti-PD-1 and anti-PD-L1 checkpoint inhibitors.^15^ Immunomodulatory effects of Gal-3 inhibition were also shown in *N*-acetyllactosamine (LacNAc; LN2; Galβ4GlcNAc) inhibitors.^16^

Poly-LacNAc-derived tetrasaccharides, presented mono- or multivalently,^17^,^18^ have proved to be very efficient inhibitors of Gal-3. These compounds exhibit affinities comparable to synthetic glycomimetics,^19^ and bypass the hurdle of limited water solubility. They are fully biocompatible and non-toxic even in high concentrations. Due to the structure of the non-reducing disaccharide motif, they are close to human milk oligosaccharides lacto-*N*-tetraose and lacto-*N*-neotetraose, known as strong ligands of Gal-3.^20^ Moreover, poly-LacNAc motifs are known to be present on the surface of many cancer cells to be recognized by galectins. While the GalNAcβ4GlcNAc (LacdiNAc; LDN) motif has been shown to be highly selective for Gal-3 compared to the other commonly occurring galectin, Gal-1,^21^ there is little information on the selectivity of other oligosaccharides from this family (Galβ4GlcNAcβ3Galβ4GlcNAc = LN2-LN2; Galβ3GlcNAcβ3GalNAcβ4GlcNAc = LN1-LN2) for Gal-3 compared to Gal-1. The previously reported poly-LacNAc-decorated structures^17,18^,^22^ were generally based on protein carriers of bovine and human serum albumins. These carriers are, however, not very prospective for any in vivo application due to their difficult preparation, relatively low stability, and limited intra-cellular penetration. Therefore, we decided to synthesize a library of the present unique tetrasaccharide structures with pronounced multivalency and increased inhibitory functions, test them in the present study, and compare them with the pilot compound GalNAcβ4GlcNAcβ3Galβ4GlcNAc (LDN-LN2). These oligosaccharides are advantageously prepared by sequential chemo-enzymatic synthesis using glycosyltransferases. Chemical methods^23^,^24^ or alternative enzymatic methods employing glycosidases have generally been shown to be less selective and lower-yielding.^25^ Due to their limited availability, the biological effects of inhibiting galectins with these compounds have not been investigated yet, with the exception of our pioneering work with LDN-LN2 tetrasaccharide.^16^

For the potential application of small molecule inhibitors in vivo, it is important to choose a suitable carrier to increase the circulation time and accumulation in the tumor tissue. The synthetic *N*-(2-hydroxypropyl)methacrylamide (HPMA) copolymers have been extensively studied as carriers of bioactive molecules since they are water-soluble, biocompatible, non-immunogenic, and non-fouling to most of the abundant proteins.^26–29^ HPMA-based polymer carriers promote the pharmacokinetics of various bioactive compounds, prolonging their circulation time in the body and increasing their uptake in specific biological targets,^26^,^27^ such as solid tumors^30^ or inflammation sites.^31^ Recently, some HPMA-based drug conjugates have already been approved for clinical trials or compassionate use.^32–34^ Our previous work demonstrated the remarkable ability of HPMA-based polymer carriers decorated with various carbohydrate epitopes to strongly bind to lectins, such as wheat germ agglutinin and galectins,^35^,^36^ and to efficiently inhibit Gal-3-induced apoptosis of T cells.^16^ By the proper selection of the type and amount of carbohydrate epitopes and linkers, we can maximize the binding efficacy of such multivalent glycopolymers and, moreover, influence the preference for Gal-1 or Gal-3.^16^,^37^,^38^ Such highly efficient glycopolymers may have multiple applications in the clinics as immunosupportive modulators of TME in current immunotherapies, such as checkpoint inhibitor therapy, antibody-drug-conjugate therapy, or adoptive immunotherapies (CAR-T, CAR-M, etc).

In this work, we synthesized different tetrasaccharide ligand motifs and their polymer conjugates with different ligand content and assessed the structure–efficiency relationship in the inhibition of Gal-1 and Gal-3. We examined the binding efficiency and selectivity of the prepared glycopolymers for Gal-1 and Gal-3 and their ability to protect J774A.1 macrophages against Gal-3-induced apoptosis. Furthermore, we tested the ability of the glycopolymers to abolish the binding of Gal-3 to IFNγ, which may lead to the scavenging of free (unbound) IFNγ in the TME.

## Materials and Methods

### Materials

1-Amino-propan-2-ol, 2,2′-azo*bis*isobutyronitrile (AIBN), 2-cyanopropan-2-yl dithiobenzoate (CTA), 2-thiazoline-2-thiol, 4-(dimethylamino)pyridine (DMAP), β-alanine, *N,N*-diisopropylethylamine (DIPEA), *N,N*-dimethylacetamide (DMA), anhydrous methanol (99.8%), *N*-ethyl-*N*′-(3-dimethylaminopropyl)carbodiimide hydrochloride (EDC.HCl), *tert*-butanol, and methacryloyl chloride were obtained from Merck (Prague, Czech Republic). Na_2_CO_3_ and NaOH were purchased from Lach-Ner (Neratovice, Czech Republic). The solvents for the nuclear magnetic resonance (NMR) characterization DMSO-*d*_6_ (99.80 atom% D) and D_2_O (99.96 atom% D) were obtained from VWR Chemicals (Radnor, USA). Purification columns (HisTrap™, MBPTrap™) were purchased from GE Healthcare (Chicago, USA). Nucleotide sugars UDP-Gal, UDP-GalNAc, and UDP-GlcNAc were prepared according to a previously published procedure.^39^ Dowex 66 free-base was obtained from Sigma Aldrich (St. Luis, USA). The competent *E. coli* cells were purchased from New England Biolabs (Ipswich, USA) or Merck (Prague, Czech Republic). Chemicals for the production of enzymes and galectins were purchased from Carl Roth GmbH (Karlsruhe, Germany). All other chemicals and solvents were of analytical grade.

### Protein Production

#### Production of Enzymes

To synthesize tetrasaccharide ligands **4** (Galβ3GlcNAcβ3Galβ4GlcNAc-*t*Boc; **LN1-LN2-*t*Boc), 5** (Galβ4GlcNAcβ3Galβ4GlcNAc-*t*Boc; **LN2-LN2-*t*Boc**), and **6** (GalNAcβ4GlcNAcβ3Galβ4GlcNAc-*t*Boc; **LDN-LN2-*t*Boc**), we produced four enzymes as N-terminal His-tagged constructs: β4-galactosyltransferase (β4GalT) from human placenta (pET16b; *Nco*I*/Xho*HI),^40^ β3-*N*-acetylglucosaminyltransferase (β3GlcNAcT) from *Helicobacter pylori* (pCWori; *Nco*I/*Xho*I),^41^ mutant human placental β4-galactosyltransferase with β4-*N*-acetylgalactosaminidase activity (β4GalT-Y284L = β4GalNAcT; pET16b; *Nco*I*/Xho*HI),^42^ and mutant β-galactosidase from *Bacillus circulans*, acting as β3-galactosynthase (BgaC-E233G; pET-Duet-1; *Bam*HI/*Pst*I).^43^ The β4-GalT and β4-GalNAcT protein constructs consisted of an N-terminal lipase pre-propeptide originating from *Staphylococcus hyicus* (202 aa; aa 39–240 of the original sequence) followed by the C-terminal GalT region with the catalytic domain (323 aa; aa 75–397 of the original sequence). The GalT construct contained one silent mutation in the nucleotide sequence, namely I210V. Two more silent mutations occurred in the GalNAcT construct (besides the target mutation Y284L); E75Q and T114A. These silent mutations did not affect catalytic abilities because they are located outside the catalytic domain. The production and purification of all enzymes were carried out as described previously; the plasmids were a kind gift from Prof. Lothar Elling, RWTH Aachen, Germany. Respective *Escherichia coli* strains (T7 Shuffle for β4GalT, β4GalNAcT; BL21(DE3) pLysS for β3GlcNAcT; BL21 Gold(DE3) for BgaC-E233G) were used for the enzyme production. The transformed cells were cultured in 60 mL of Luria Bertani (LB) medium (10 g/L tryptone, 5 g/L yeast extract, 5 g/L NaCl) containing respective antibiotics (according to the used *E. coli* strain and plasmid) in 0.5 L flasks at 37 °C and 220 rpm overnight. This pre-culture was then used to inoculate 600 mL of Terrific Broth (TB) medium (24 g/L yeast extract, 12 g/L tryptone, 4 mL/L glycerol, 17 mM KH_2_PO_4_, 72 mM K_2_HPO_4_, pH 7.5) containing antibiotics in 3 L flasks, and grown at 37 °C and 150 rpm until optical density OD_600_ reached 0.6–0.8. Then, enzyme expression was induced by 0.5 mM IPTG, and the cultures were grown for 24 h at 25 °C and 150 rpm. The cells were harvested by centrifugation at 8,880 × g for 20 min at 4 °C, and stored at −20 °C. For purification, the cells were sonicated (1 min pulse, 2 min pause, 6 cycles) in the respective equilibration buffer (see below). The cell-free extract was then loaded onto an equilibrated affinity chromatography column. The N-terminal His-tagged protein constructs (β4GalT, β4GalNAcT, BgaC-E233G) were purified on a HisTrap™ column with equilibration buffer (20 mM phosphate/500 mM NaCl/20 mM imidazole, pH 7.4) and eluted by enhanced concentration of imidazole (20 mM phosphate/500 mM NaCl/500 mM imidazole, pH 7.4). The maltose-binding-protein (MBP)-tagged protein construct β3GlcNAcT was purified on an MBPTrap™ column with equilibration buffer (20 mM Tris-HCl/200 mM NaCl/1 mM EDTA, pH 7.4) and eluted by maltose (10 mM) in the binding buffer. In some reactions, crude β4GalT without purification could be used to enhance the enzyme yield. All enzymes were dialyzed overnight against PBS buffer and their concentration was determined by Bradford assay (calibrated for IgG).

#### Enzyme Activity Assay

The catalytic activity of recombinant glycosyltransferases was determined as follows: The respective UDP-sugar glycosyl donor (UDP-GlcNAc for β3GlcNAcT, UDP-Gal for β4GalT, UDP-GalNAc for β4GalNAcT; 6.5 mM) was mixed with the respective glycosyl acceptor (LacNAc-*t*Boc for β3GlcNAcT, GlcNAc-*t*Boc or GlcNAc-LacNAc-*t*Boc for β4GalT, GlcNAc-LacNAc-*t*Boc for β4GalNAcT; 5 mM), lactate dehydrogenase (20 U/mL), pyruvate kinase (20 U/mL), NADH (0.25 mM), and phosphoenolpyruvate (1 mM) in 100 mM HEPES/25 mM KCl buffer pH 7 with MgCl_2_ (4 mM) for GlcNAcT or MnCl_2_ (4 mM) for GalNAcT. After 5 min pre-incubation at 37 °C, the respectively diluted enzyme was added (final reaction volume 100 µL). The reaction was monitored for 15 min, and the linear decline in NADH concentration from the coupled reaction was monitored in time at 340 nm.

#### Production of Galectins

Recombinant human galectins Gal-1 and Gal-3 were produced as N-terminal His-tagged protein constructs cloned into pET-Duet1 vector using *Nco*I/*Asc*I restriction sites as previously described.^17^,^19^ For Gal-1, the mutant variant C2S was used to prevent oxidation of the protein.^44^ Briefly, *E. coli* Rosetta 2(DE3)pLysS competent cells were transformed with the respective plasmid and cultured overnight at 37 °C and 220 rpm in 60 mL LB medium with ampicillin (100 µg/mL) and chloramphenicol (34 µg/mL). The preculture was then inoculated into 600 mL of TB medium, supplemented with the same antibiotics, and grown at 37 °C and 120 rpm. When the culture reached OD_600_ of 0.6–0.8, 0.5 mM IPTG was used for inducing the protein expression, followed by cultivation at 25 °C, 140 rpm for 24 h. The production of biotinylated Gal-3 was accomplished by a previously described method.^17^ The Gal-3-AVI construct carrying an AVI-tag (15-amino-acid sequence containing one lysine residue selective for monobiotinylation: GLNDIFEAQ**K**IEWHE) in the vector pET-Duet1 was expressed in *E. coli* BL21 (DE3) (Takara Bio, Kusacu, Japan) containing an IPTG-inducible plasmid with the gene *birA* of biotin ligase. The cells were cultured at 37 °C and 140 rpm in MDO medium (20 g/L yeast extract, 20 g/L glycerol, 1 g/L KH_2_PO_4_, 3 g/L K_2_HPO_4_, 2 g/L NH_4_Cl, 0.5 g/L Na_2_SO_4_), supplemented with ampicillin (150 µg/mL), chloramphenicol (10 µg/mL) until OD_600_ reached 0.6. Then, d-biotin (50 µM final concentration) was added, and the protein expression was induced by IPTG (1 mM). Cultures were grown for 4 h more at 37 °C, and harvested.

For the purification of all galectins, the harvested cells (8,880 × g, 20 min, 4 °C) were sonicated in an equilibration buffer (20 mM phosphate/500 mM NaCl/20 mM imidazole, pH 7.4) for 6 cycles of 1 min pulse and 2 min pause to disrupt the cells. The cell-free extract was then loaded onto an equilibrated Ni-NTA column (GE Medical Systems, Prague, Czech Republic), and the loaded column was washed successively with equilibration buffer (20 mM phosphate/500 mM NaCl/20 mM imidazole, pH 7.4). Then, the column was washed with equilibration buffer containing 0.5% Triton X-100 to remove lipopolysaccharide^19^ and again with pure equilibration buffer. The protein elution was accomplished by elution buffer containing 500 mM imidazole. The eluted fractions were analyzed for protein content using the Bradford assay (calibrated for bovine serum albumin, BSA), pooled and dialyzed overnight in PBS buffer (20 mM phosphate/150 mM NaCl, pH 7.5; 7 L) containing 2 mM EDTA (ethylenediaminetetraacetic acid), followed by 4 h dialysis in PBS buffer (7 L). The recombinant galectins were stable at 4 °C for several months.

### Synthetic Procedures

#### Synthesis of Functionalized Tetrasaccharide Ligands

##### General Procedure for the Synthesis of Tetrasaccharides 4 (LN1-LN2-*t*Boc), 5 (LN2-LN2-*t*Boc), 6 (LDN-LN2-*t*Boc)

The synthetic precursor, (*tert*-butoxycarbonylamino)ethylthioureidyl 2-acetamido-2-deoxy-β-d-glucopyranoside (GlcNAc-*t*Boc, **1**), was prepared from *N*-acetylglucosamine in a synthetic procedure described previously.^45^ In all enzymatic steps with glycosyltransferases, the respective glycosyl acceptor (GlcNAc-*t*Boc **1** or GlcNAc-LacNAc-*t*Boc **3**, 5 mM) was mixed with the glycosyl donor (UDP-GlcNAc or UDP-Gal or UDP-GalNAc, 6.5 mM) and suspended in the respective buffer. Then, the respective glycosyltransferase was added, and the reactions were incubated overnight at 37 °C under shaking at 300 rpm. For the synthesis of tetrasaccharide **4** (LN1-LN2-*t*Boc) we used GlcNAc-LN2-*t*Boc trisaccharide acceptor (**3**, 10 mM), which was glycosylated using α-d-galactosyl fluoride (α-Gal-F) as a donor under the catalysis by mutant glycosidase BgaC-E233G. All enzymatic reactions were stopped by thermal enzyme inactivation at 99 °C for 5 min, and the enzyme-free reaction mixture was purified by gel permeation chromatography (GPC; Biogel P2, 1,000 × 30 mm, H_2_O mobile phase; 6.5 mL/h flow rate). In case the final products were not 100% pure after size exclusion chromatography, they were re-purified by HPLC (reversed-phase analytic MultoKrom 100–5 C18 column; 250 × 4.6 mm; CS Chromatographie, Langerwehe, Germany) with 85/15 *v*/*v* H_2_O/acetonitrile as a mobile phase, at a flow rate of 1 mL/min, and detection at 220 nm. The fractions containing the pure product were pooled and lyophilized.

##### (*tert*-Butoxycarbonylamino)ethylthioureidyl 2-Acetamido-2-Deoxy-β-D-Glucopyranosyl-(1→3)-β-D-Galactopyranosyl-(1→4)-2-Acetamido-2-Deoxy-β-D-Glucopyranoside (3; GlcNAc-LN2-*t*Boc)

Trisaccharide **3** was produced from GlcNAc-*t*Boc (**1**) by sequential glycosylation catalyzed by human placental β4GalT^40^ and *H. pylori* β3GlcNAcT^41^ in two consecutive reactions. In the first step, crude β4GalT (0.03 U/mL in the reaction) originated by resuspending 10 g of T7 shuffle *E. coli* cells (WCW) in 20 mL of 50 mM sodium phosphate buffer pH 7.4, sonication (6 cycles of 1 min pulse and 2 min pause) and filtration (0.8 µm), was incubated with UDP-Gal (295 mg, 6.5 mM) and acceptor **1** (170 mg, 5 mM) in 100 mM sodium phosphate/25 mM KCl/2 mM MnCl_2_ pH 7 (total reaction volume 80 mL) overnight at 37 °C. To reach complete conversion, the reaction was re-fed with the same amount of crude β4GalT and 5 mM UDP-Gal (227 mg) after 24 h. The reaction was monitored by TLC (isopropyl alcohol/H_2_O/NH_4_OH aq.; 7/2/1 *v*/*v*/*v*) to confirm the complete conversion of acceptor **1** to LN2-*t*Boc **2**. Product **2** was purified by GPC as described in the General procedure, resulting in 140 mg (60% isolated yield) of LN2-*t*Boc **2**. In the next step, β3GlcNAcT (0.02 U/mL in the reaction) catalyzed the glycosylation of LN2-*t*Boc **2** (140 mg, 5 mM) acceptor using UDP-GlcNAc (197 mg, 6.5 mM) as a donor in 100 mM sodium phosphate/25 mM KCl/5 mM MgCl_2_/1 mM DTT buffer, pH 7 (total reaction volume 50 mL). After confirming complete conversion by TLC, product **3** was purified by gel chromatography as above, resulting in 160 mg of pure **3** (85% yield). The structure of **3** was confirmed by NMR (Supporting Information, Table S1, Figures S1A, S1B). HRMS and HPLC characterizations are shown in the Supporting Information, Figures S1C and S1D.

##### (*tert*-Butoxycarbonylamino)ethylthioureidyl β-D-Galactopyranosyl-(1→3)-2-Acetamido-2-Deoxy-β-D-Glucopyranosyl-(1→3)-β-D-Galactopyranosyl-(1→4)-2-Acetamido-2-Deoxy-β-D-Glucopyranoside (4; Galβ3GlcNAcβ3Galβ4GlcNAc-*t*Boc; LN1-LN2-*t*Boc)

Galactosyl donor α-Gal-F (18.2 mg, 10 mM), prepared as described previously,^46^ and acceptor GlcNAc-LN2-*t*Boc (**3**; 79 mg, 10 mM) were dissolved in 50 mM sodium phosphate buffer pH 6.5, and β3-galactosynthase BgaC-E233G (74 mg, 559 µM, 5270 mL) was added (total reaction volume 10 mL). The reaction was incubated at 30 °C and 850 rpm and monitored by TLC. When all donor was consumed (ca after 20–24 h), the reaction was stopped by enzyme denaturation and purified by gel chromatography, as described in the General procedure, affording 80 mg of **4** (84% yield). To reach an even higher purity required for multivalent conjugation, product **4** was optionally purified by HPLC as described in the General procedure. The structure of **4** was confirmed by NMR and was in accord with the published data.^18^ HRMS and HPLC characterizations are shown in the Supporting Information, Figures S2A and S2B.

##### (*tert*-Butoxycarbonylamino)ethylthioureidyl β-D-Galactopyranosyl-(1→4)-2-Acetamido-2-Deoxy-β-D-Glucopyranosyl-(1→3)-β-D-Galactopyranosyl-(1→4)-2-Acetamido-2-Deoxy-β-D-Glucopyranoside (5; Galβ4GlcNAcβ3Galβ4GlcNAc-*t*Boc; LN2-LN2-*t*Boc)

UDP-Gal (74 mg, 6.5 mM) as a donor and trisaccharide **3** (79 mg, 5 mM) as an acceptor were combined with crude β4GalT (0.03 U/mL in the reaction) in 100 mM sodium phosphate/25 mM KCl/2 mM MnCl_2_ buffer, pH 7 (total reaction volume 20 mL). The conversion monitored by TLC was 100%. Purification by GPC (see the General procedure) yielded 90 mg of product **5** (94% yield), which was optionally additionally purified by HPLC. The structure of **5** was confirmed by NMR and was in accord with the published data.^47^ HRMS and HPLC characterizations are shown in the Supporting Information, Figures S3A and S3B.

##### (*tert*-Butoxycarbonylamino)ethylthioureidyl 2-Acetamido-2-Deoxy-β-D-Galactopyranosyl-(1→4)-2-Acetamido-2-Deoxy-β-D-Glucopyranosyl-(1→3)-β-D-Galactopyranosyl-(1→4)-2-Acetamido-2-Deoxy-β-D-Glucopyranoside (6; GalNAcβ4GlcNAcβ3Galβ4GlcNAc-*t*Boc; LDN-LN2-*t*Boc)

UDP-GalNAc (150 mg, 6.5 mM) as a donor was combined with trisaccharide **3** (150 mg, 5 mM) as an acceptor and β4GalNAcT (0.03 U/mL of the reaction mixture) in 100 mM sodium phosphate/25 mM KCl/2 mM MnCl_2_ buffer, pH 7 (total reaction volume 38 mL). The reaction was re-fed twice (on day 2 and day 3) with the same amounts of enzyme and UDP-GalNAc as used in the initial reaction. The final conversion of tetrasaccharide **6** was 40%. After purification by GPC (see the General procedure) the yield was 40 mg, 22%. To reach an ultimate purity, product **6** was subsequently purified by HPLC (see the General procedure). The structure of **6** was confirmed by NMR and was in accord with the published data.^18^ HRMS and HPLC characterizations are shown in the Supporting Information, Figures S4A and S4B.

##### Deprotection of Tetrasaccharides 4, 5, and 6

To obtain free amines **4a, 5a**, and **6a** for conjugation to copolymer carriers, *t*Boc-capped tetrasaccharides **4, 5**, and **6** (10 mM) were deprotected in 1 M HCl. The reaction mixtures were incubated overnight at 4 °C. The complete conversion to the deprotected sugars was confirmed by TLC (isopropyl alcohol/H_2_O/NH_4_OH aq; 7/2/1 *v*/*v*/*v*). The reactions were neutralized by dilution with water and the addition of Dowex 66 base-free. Deprotected glycans **4a, 5a**, and **6a** were subsequently lyophilized (85–95% yield after amine deprotection).

#### Synthesis of Glycopolymers

Monomers *N*-(2-hydroxypropyl)methacrylamide (HPMA) and *N*-methacryloyl-β-alanine thiazolidine-2-thione (MA-AP-TT) were synthesized according to the literature.^48^,^49^ Their purity was assessed using HPLC, elemental analysis, and nuclear magnetic resonance (^1^H NMR in DMSO-*d_6_*).

The polymer precursors **7** and **8** bearing TT groups poly(HPMA-*co*-MA-AP-TT) were prepared by the controlled radical reversible addition-fragmentation chain transfer (RAFT) copolymerization of HPMA and MA-AP-TT in a mixture of *tert*-butanol and DMA (8/2, *v/v*), using adopted conditions from our previous work.^37^ A detailed synthetic procedure is described in Supplementary Material 1.

**Scheme 1:**
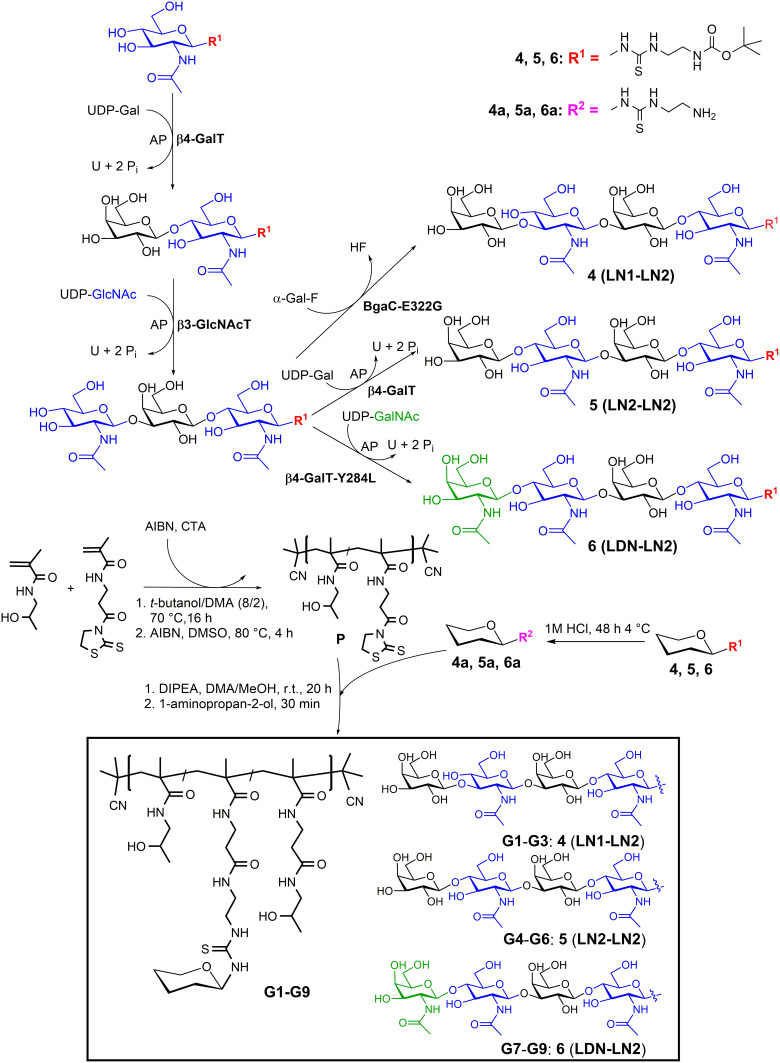
Sequential enzymatic synthesis of functionalized tetrasaccharide ligands: **4** (**LN1-LN2**), **5** (**LN2-LN2**), **6** (**LDN-LN2**) followed by the synthesis of polymer precursors and glycopolymers **G1–G9**.

The glycopolymers **G1–G9** were prepared by aminolysis of TT groups of the polymer precursors with the corresponding amount of amino-functionalized tetrasaccharides **LN1-LN2** (forming glycopolymers **G1–G3), LN2-LN2** (forming glycopolymers **G4–G6**), or **LDN-LN2** (forming glycopolymers **G7–G9**), using DIPEA (see Scheme 1). Reaction conditions were adopted from our previous work.^37^ See Supplementary Material 1 for a detailed synthetic procedure. The physicochemical characterization of the polymer precursors and glycopolymers **G1–G9** is shown in Supporting Information, Table S2.

### Analytical Methods

#### High-Performance Liquid Chromatography

The purity of tetrasaccharides **4, 5**, and **6** was analyzed using Shimadzu Prominence LC analytical system with hydrophilic interaction liquid chromatography (HILIC) column TSKgel Amide-80, 5 µm (Tosoh Bioscience, DE) and detection at 200 nm as described previously.^37^ The purity of the monomers HPMA and MA-AP-TT, and the course of aminolysis reactions to obtain glycopolymers **G1–G9** were monitored using a Shimadzu HPLC system equipped with a C18 reversed-phase Chromolith Performance RP-18e column (150 mm) and a diode array detector (Shimadzu SPD-M20A). Water/acetonitrile/0.1% TFA with a gradient of 5−95%, *v/v* acetonitrile at a flow rate of 4 mL/min was used as an eluent.

#### Dynamic Light Scattering

The hydrodynamic diameter (*D*_h_) of polymer precursors and glycopolymers **G1–G9** was measured by dynamic light scattering (DLS) using a Nano-ZS instrument (ZEN3600, Malvern, UK) at *λ* = 632.8 nm and *θ* = 173°. Samples were prepared in PBS (pH 7.4) at 3 mg/mL using a 0.22 µm polyvinylidene fluoride (PVDF) filter. The values were determined as a mean of at least five independent measurements, and all data were evaluated using DTS (Nano) software.

#### UV–Vis Spectrophotometry

The molar content of thiazolidine-2-thione (TT) groups statistically distributed along the polymer precursors was assessed using a Specord 205 ST spectrophotometer (Analytic Jena AG, Germany). The molar absorption coefficient was *ε*(TT) = 10,300 L/mol/cm in methanol (*λ*_max_ = 305 nm).

#### Gel Permeation Chromatography

The number-average molecular weight (*M*_n_), and the dispersity (*Đ*) of the polymer precursors and glycopolymers **G1–G9** were determined using a Shimadzu HPLC system equipped with GPC columns. For the precursors and glycopolymers **G1-G2, G4-G5**, and **G7-G8**, a TSKgel Super SW3000 (Tosoh Bioscience, Tokyo, Japan) (300 × 4.6 mm, 4 μm) column was used. The analysis was performed in a mixture of methanol and 0.3 M sodium acetate buffer at pH 6.5 (8/2, *v*/*v*) as a mobile phase at a flow rate of 0.3 mL/min. Glycopolymers with a high content of carbohydrate ligands **G3, G6**, and **G9** were analyzed on a Superose^TM^ 6 Increase (300 × 10 mm, 8.6 μm) column using 0.2 M phosphate buffer at pH 7.4 as a mobile phase at a flow rate of 0.5 mL/min. A differential refractive index (RI) (Optilab-rEX, Wyatt Technology Co., USA), a multi-angle light scattering (MALS) (DAWN HELEOS II, Wyatt Technology Co., USA), and an SPD-M20A photodiode array (Shimadzu, Japan) detectors were used. All data were analyzed using the ASTRA VI software, and the refractive index increment d*n*/d*c* (≈ 0.167 mL/g) was directly determined using the RI detector (with 100% recovery of the injected sample from the column) and used for the calculation of *M*_n_, and *Ð*.

#### Nuclear Magnetic Resonance

^1^H and ^13^C NMR analyses of compounds **1–6**, including COSY, 1D-TOCSY, HSQC, and HMBC, were performed on Bruker AVANCE III 400 and 700 MHz spectrometers (Bruker BioSpin, Rheinstetten, Germany) in D_2_O at 30 °C. Residual signals of D_2_O (*δ*_H_ 4.73 ppm) or acetone signal (*δ*_c_ 30.50) were used for referencing. The obtained NMR data were analyzed using TopSpin 3.5 software and were consistent with the previously reported values.^17^,^18^ A Bruker AVANCE III 600 spectrometer operating at 600.2 MHz was used to measure ^1^H NMR spectra of monomers (in DMSO-*d*_6_) and glycopolymers **G1–G9** (in D_2_O). NMR spectra were recorded in 5 mm tubes, and the conditions for measurements were as follows: π/2 pulse width 18 μs, relaxation delay 10s, spectral width 15 kHz, acquisition time 2.18 s, and 16−200 scans. The integral intensity of the signals at *δ* (ppm) ≈ 3.07 and 3.19 (2H, C*H*_2_–NH) of the HPMA unit was applied. The molar content of **4a** (**LN1-LN2**) and **5a** (**LN2-LN2**) in glycopolymers **G1–G3** and **G4–G6**, respectively, was calculated using the integral intensity of signals at *δ* (ppm) ≈ 4.48 (1H, C*H* of C-1 of *N*-acetylated Glc^C^), *δ* (ppm) ≈ 4.44 (1H, C*H* of C-1 of Gal^B^), and *δ* (ppm) ≈ 4.16 (1H, C*H* of C-1 of Gal^D^). The molar content of **6a** (**LDN-LN2**) in glycopolymers **G7–G9** was calculated as previously reported,^17^ using the integral intensity of signals at *δ* (ppm) ≈ 4.49 and 4.51 (1H, C*H* of C-1 of *N*-acetylated Gal^D^), *δ* (ppm) ≈ 4.45 (1H, C*H* of C-1 of Gal^B^), and *δ* (ppm) ≈ 4.12 (1H, C*H* of C-4 of Gal^B^). Representative spectra are shown in the Supporting Information, Figures S5A–C.

#### High-Resolution Mass Spectrometry

The electrospray mass spectra were measured with an LTQ Orbitrap XL hybrid mass spectrometer (Thermo Fisher Scientific, Waltham, USA), using methanol/water (4:1, *v*/*v*) as a mobile phase (flow rate of 100 μL/min). The sample was dissolved in water and injected into the mobile phase flow using a 10 μL loop. Data were processed with Xcalibur Software (Thermo Fisher Scientific). For the positive ion mode, spray voltage, capillary voltage, tube lens voltage, and capillary temperature were 5.0 kV, 9 V, 150 V, and 275 °C, respectively. The Orbitrap mass spectra were recorded at the resolution of 100,000.

### Biological Evaluation

#### Cell Line

Murine macrophages J774A.1 were purchased from Sigma-Aldrich (St. Louis, MO, USA). The cells were cultivated in a complete growth medium containing DMEM (Gibco) supplemented with 1% penicillin–streptomycin (Thermo Fisher Scientific, Waltham, MA, USA) and 10% heat-inactivated fetal bovine serum (FBS; Thermo Fisher Scientific). The cell line was grown in a humidified 5% CO_2_ atmosphere at 37 °C.

#### Inhibition of Galectin Binding by Glycopolymers (Competitive ELISA-Type Assay)

The affinity of glycopolymers **G1-G9** to galectins Gal-1 and Gal-3 was determined by employing a competitive ELISA assay with immobilized asialofetuin (ASF) as the competitive ligand, as described previously.^18^ Microtiter plates (Nunc Immuno Sorb, ThermoFisher Scientific, USA) were coated with ASF (0.1 µM in PBS, pH 7.5, 50 µL/well) and incubated overnight. The wells were washed with 3×250 µL of PBS containing 0.05% Tween (repeated after each step). The wells were then blocked by 2% *w/v* BSA in PBS buffer (250 µL/well) for 1 h. Serial dilutions of the ligands (glycopolymers or oligosaccharides) in EPBS (2 mM EDTA in PBS) were incubated with Gal-1 or Gal-3 (1.25 µM final concentration) for 2 h. The residual bound galectin was labeled with anti-His antibody conjugated to horseradish peroxidase (Santa Cruz; 1:1000 dilution in PBS, 1 h). TMB One substrate solution (Kem-En-Tech, Denmark; 50 µL/well) was added, and the reactions were incubated until visible blue staining appeared (3–20 min). The reaction was stopped by adding 3 M HCl (50 µL per well), resulting in a color shift to yellow, which was determined spectrophotometrically by an absorbance microplate reader (Sunrise Tecan Group Ltd, CH). The intensity of the signal at 450 nm corresponded to the amount of galectin bound to the wells. Non-linear regression (dose–response inhibition-variable slope) of the sigmoidal curves was used to calculate the values of half-maximal inhibitory constants (IC_50_) from at least six independent experiments using GraphPad Prism 7 (GraphPad Software, USA).

#### Cytotoxicity Evaluation of Glycopolymers

For the cytotoxicity evaluation of prepared glycopolymers, undifferentiated J774A.1 macrophages were seeded in a concentration of 2×10^4^ cells per well in 100 µL of complete growth medium. Cells were grown for 24 hours and then treated with glycopolymers in a final concentration of 0–80 µM. After 24 hours of incubation, a cell counting kit-8 (CCK-8) assay was added to the cells according to the manufacturer’s instructions (10 µL of CCK-8 to 100 µL of medium) and incubated at 37 °C for 3 hours. The absorbance was measured at wavelength 450 nm, and the viability of cells in each well was calculated in comparison to control. Each experiment was performed in triplicate in two independent experiments.

#### Flow Cytometry

For flow cytometry studies, cultured undifferentiated (without any LPS and cytokines) J774A.1 murine macrophages were seeded in a concentration of 10^5^ cells/well of the 24-well plate. After 24 h of cultivation, cells were pretreated with the desired concentration of glycopolymers for 5 minutes and afterward treated with 10 μM Gal-3 for 24 h. After harvesting of cells, early and late apoptoses of Gal-3-treated J774A.1 cells in the presence or absence of glycopolymers were evaluated using eBioscience Annexin V apoptosis detection kit (Thermo Fisher Scientific). Flow cytometry was performed using FACS Canto II (BD Biosciences, Franklin Lakes, NJ, USA). Apoptotic half-maximal inhibitory concentrations (IC_50_) of individual glycopolymers were calculated by nonlinear regression of log(glycopolymer concentration) versus apoptosis response using GraphPad Prism 8 software (version 8.0.1; GraphPad Software, La Jolla, CA, USA).

#### Galectin-3-Coated Beads and ELISA Assay

The saturating concentration of recombinant human Gal-3 biotinylated at lysin of the N-terminal AVI-tag (prepared as described in Bumba et al, 2018)^17^ was incubated with Dynabeads M-280 Streptavidin (Thermo Fisher Scientific) overnight at 4 °C. After incubation, beads were washed three times with PBS containing 2 mM EDTA and 0.05% *v/v* Tween 20. Gal-3-coated beads in the Gal-3/IFNγ molar ratio 1:10 were incubated in the presence or absence of glycopolymers and with glycosylated IFNγ (Sigma-Aldrich) at gentle shaking for 1 h at room temperature. Then, Gal-3-coated Dynabeads were magnetically separated from the samples, and IFNγ present in supernatants was measured by human IFNγ-detecting ELISA (Thermo Fisher Scientific).

#### Statistical Analysis

Statistical significance was evaluated by one-way analysis of variance (ANOVA) with Dunnett’s post-hoc test. Cell culture data were performed in duplicates of at least two independent experiments (n ≥ 2). Graphs and statistical analysis were performed in GraphPad Prism software (version 8.0.1; GraphPad Software, La Jolla, CA, USA). A *P*-value of <0.05 was considered as significant.

## Results and Discussion

### Synthesis of Functionalized Tetrasaccharide Ligands

A selection of tetrasaccharide ligands of poly-LacNAc type, capped with different terminal carbohydrate units: β(1→3)-bound d-Gal in **LN1-LN2** (**4**), β(1→4)-bound d-Gal in **LN2-LN2** (**5**) or β(1→4)-bound d-GalNAc in **LDN-LN2** (**6**) were prepared to assess the impact of their differing structure and multivalent presentation on their affinity and selectivity to Gal-3. The ligand choice was based on previous reports,^16^,^17^ which, however, never addressed the selectivity issue. Sufficient selectivity (at least 100-fold) is crucial to target one particular galectin in a real situation where, commonly, a whole battery of galectins (at least Gal-1 and Gal-3) is naturally expressed. Notably, their biological functions can sometimes be contradictory, especially in processes as complex as carcinogenesis and immunomodulation. The synthetic strategy affording functionalized tetrasaccharide ligands **4, 5**, and **6** involved a stepwise enzymatic glycosylation of a chemically functionalized precursor GlcNAc-*t*Boc^45^ as outlined in Scheme 1. For this aim, a library of enzymes – glycosyltransferases β4GalT, mutant β4GalT-Y284L with β4GalNAcT activity, β3GlcNAcT, and mutant β3-galactosynthase BgaC-E233G – were heterologously produced in appropriate *E. coli* strains, and purified by affinity chromatography (HisTrap™ or MBPTrap™ column, depending on the particular protein construct). The glycosylations catalyzed by glycosyltransferases were accomplished using respective sugar nucleotides (UDP-Gal, UDP-GalNAc, or UDP-GlcNAc)^40^ as glycosyl donors, whereas the galactosynthase-catalyzed reaction employed α-d-galactosyl fluoride donor.^46^ All donors were prepared using the previously described synthetic procedures.^38^,^46^ Though reaction conversions were generally close to quantitative, losses were encountered during product purification by GPC, reaching isolated yields of 60–93%. The only exception was mutant β4GalT = β4-GalNAcT, the least stable enzyme from the series, with which it is very challenging to reach high conversion rates (the isolated yield of this step was 21%). The glycosyltransferase-catalyzed reactions are generally burdened by high concentrations of buffer salts required for a good catalytic function. Some of these salts, such as HEPES,^18^ may even extremely complicate purification. Therefore, we optimized the synthetic pathways to minimize this problem in the present procedures. If the purification by gel chromatography was still insufficient to reach the supreme purity of samples, HPLC purification was optionally applied as described in the Materials and Methods Section. After *t*Boc deprotection, amines **4a, 5a**, and **6a** were used for conjugation to HPMA-based copolymers.

### Synthesis and Physicochemical Characterization of Glycopolymers

The controlled RAFT polymerization technique was used to prepare the polymer precursors with *M*_n_ about 20 and 30 kg/mol, a narrow dispersity (*Đ* = 1.1), and *D*_h_ around 7 nm. Such design ensures their further safe elimination from the organism due to the limit of glomerular filtration^50^ for linear HPMA-based copolymers. The amount of TT groups (about 13 mol. %) statistically distributed along the polymer backbone was sufficient for the covalent attachment of the tetrasaccharide ligands in different amounts (denoted as very low, low, and high content, see Table 1 and Table S2 in the Supporting Information). The prepared HPMA-based glycopolymers carried from ca. 3 to 9 mol. % of the amino-functionalized tetrasaccharide **4a** (**LN1-LN2**) affording glycopolymers **G1–G3**, tetrasaccharide **5a** (**LN2-LN2**) affording glycopolymers **G4–G6**, or tetrasaccharide **6a** (**LDN-LN2**) affording glycopolymers **G7–G9** (see Scheme 1). As expected, the molecular weights of glycopolymers were increasing with the increasing content of tetrasaccharide ligands in the glycopolymers (see Supporting Information, Table S2). Their dispersity did not increase, indicating an absence of cross-linking reactions. In addition, the presence of ligands attached to the polymer carrier did not cause an increase in the hydrodynamic diameters.

**Table 1:** Inhibitory Potency of Glycopolymers to Gal-3

Cmp	Glycan Motif ^a^	n Glycans ^b^	Glycan Content [mol. %] ^c^	IC_50_ [µM]/Glycan (*rp**/**n*) ^d^	IC_50_ [µM] ^e^ (*rp*) ^f^	Selectivity for Gal-3^g^
**4**	LN1-LN2-*t*Boc	1	-	27.1 ± 4.4	27.1 ± 4.4	17
**G1**	LN1-LN2 very low	5.3	2.7	1.0 ± 0.30 *(25)*	0.189 ± 0.056 (*131*)	140
**G2**	LN1-LN2 low	6.5	4.0	1.0 ± 0.54 *(25)*	0.153 ± 0.084 (*156*)	17
**G3**	LN1-LN2 high	15.1	7.8	2.7 ± 1.1 *(9)*	0.179 ± 0.073 (*139*)	8.9
**5**	LN2-LN2-*t*Boc	1	-	10.2 ± 3.2	10.2 ± 3.2	10
**G4**	LN2-LN2 very low	2.2	1.8	1.43 ± 0.53 *(9)*	0.65 ± 0.82 (*20*)	190
**G5**	LN2-LN2 low	7.1	4.4	1.50 ± 0.55 *(9)*	0.211 ± 0.078 (*62*)	150
**G6**	LN2-LN2 high	9.9	7.9	0.22 ± 0.11 *(59)*	0.022 ± 0.011 (*591*)	280
**6**	LDN-LN2-*t*Boc	1	-	7.4 ± 0.5	7.4 ± 0.5	13
**G7** ^h^	LDN-LN2 very low	3.4	2.6	0.22 ± 0.06 *(32)*	0.066 ± 0.018 (*106*)	> 900
**G8** ^h^	LDN-LN2 low	5.9	4.9	0.14 ± 0.05 *(50)*	0.023 ± 0.009 (*304*)	> 1800
**G9** ^h^	LDN-LN2 high	11.1	9.7	4.4 ± 1.0 *(1.6)*	0.40 ± 0.09 (*18*)	> 68

**Notes**: ^a^ **LN1-LN2**, Galβ3GlcNAcβ3Galβ4GlcNAc; **LN2-LN2**, Galβ4GlcNAcβ3Galβ4GlcNAc; **LDN-LN2**, GalNAcβ4GlcNAcβ3Galβ4GlcNAc. ^b^ *n*, the average number of glycan ligands per polymer chain, ^c^ content of glycan ligands in mol. %; n = 1, monovalent oligosaccharide (free sugar standard). ^d^ *rp*/*n* means relative potency per one glycan ligand presented on the glycopolymer, ie, IC_50_ of the respective monovalent oligosaccharide/IC_50_ per glycan of the glycopolymer.^e^ IC_50_ (half-maximal inhibitory potency) is the concentration of glycopolymer required to inhibit Gal-3 binding to immobilized ASF by 50%. ^f^ *rp* means relative potency, ie, IC_50_ of the respective monovalent oligosaccharide/IC_50_ of the glycopolymer. ^g^ Selectivity for Gal-3 (IC_50_ for Gal-1/IC_50_ for Gal-3). Values for Gal-1 are stated in the Supporting Information, Table S3. ^h^ Data were adopted from our previous study.^16^ IC_50_ of lactose was 116 ± 26 µM for Gal-3 and 310 ± 38 µM for Gal-1.

### Affinity of Glycopolymers to Galectins

To determine the inhibitory potency of prepared glycopolymers **G1-G9**, we produced human Gal-3 as a recombinant His-tagged protein construct in *E. coli* Rosetta 2(DE3)pLysS as described previously.^17^,^19^ For the affinity measurement, the competitive ELISA assay with ASF as an immobilized competitor ligand was employed.^51^ Various concentrations of glycopolymers inhibited the binding of galectins to ASF. The inhibitory potency of the prepared glycopolymers was compared to the respective monovalent standards (LN1-LN2-*t*Boc, LN2-LN2-*t*Boc, LDN-LN2-*t*Boc) to calculate their relative potencies (*rp*) for inhibition of Gal-3 (Table 1). Relative potency values thus disclose the extent of the so-called multivalency effect of the glycopolymer – the ability of a ligand bound to a multivalent carrier to get easier access and orientation to the lectin-binding site.^52^

**Figure 1:**
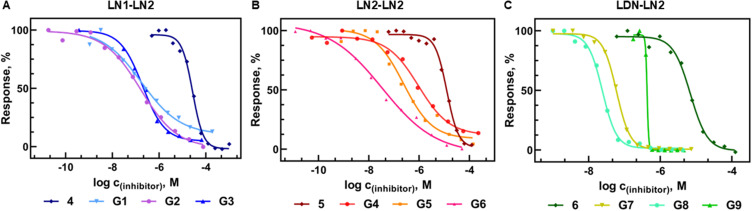
Competitive inhibition of binding of Gal-3 to ASF by glycopolymers determined by ELISA. Dose-response inhibition curves for **LN1-LN2**-based (**A**), **LN2-LN2**-based (**B**), and **LDN-LN2**-based (**C**) glycopolymers. IC_50_ values are stated in Table 1.

The affinity trends differed across the tested series (Figure 1) but, generally, all tetrasaccharide ligands, even monovalent, exhibited a greatly enhanced performance compared with a simple disaccharide ligand lactose (IC_50_ = 116 ± 26 µM) due to their ability to address the whole spectrum of binding subsites A-E in the galectin-binding groove. Whereas with the **LN1-LN2** motif, there were negligible differences in affinity between the very low, low, and high content of glycan, significant differences were found in the other two series. The **LN2-LN2** motif showed the highest efficiency with the densest presentation of 8 mol. % (**G6**; IC_50_ = 22 nM). In the **LDN-LN2** series, the most advantageous presentation was 5 mol. % (**G8**; IC_50_ = 23 nM). In this case, a denser glycan presentation showed lower affinity, probably due to the sterical hindrance in the Gal-3 binding site. Here, the “high” ligand content was the highest of all tested glycopolymers, reaching 10 mol. %. The differences in affinity between different polymer-bound oligosaccharides were generally not very large. However, the multivalent effect caused an affinity increase of two orders of magnitude in glycopolymers compared to the respective low-molecular-weight ligands. Nevertheless, the density of multivalent presentation had a certain impact; the most efficient tetrasaccharide ligands content in terms of maximum affinity appeared to be around 5–8 mol. % of LDN-LN2. Furthermore, we did not observe any difference between polymer precursors of 20 or 30 kg/mol. We conclude that the most important parameter is the density of the ligands across the polymer chain, not the length of the polymer chains in such a short size window.

As for the selectivity of inhibition compared to the other abundant galectin Gal-1 (Supporting Information, Table S3), the increase in affinity with the increasing glycan content was a clear trend that applied to all glycan motifs. We previously observed such behavior in glycopolymers bearing *N*‑acetyllactosamine.^37^ For example, glycopolymer **G6** with 9.9 glycans had a 20-times higher affinity to Gal-1 than glycopolymer **G4** with 2.2 glycans. Notably, all the prepared glycopolymers were selective inhibitors of Gal-3 (up to over 1800-fold higher affinity to Gal-3 over Gal-1; cf. Table 1 and Table S3 in Supporting Information). We have already shown previously^16^,^22^,^38^ that *N,N’*-diacetyllactosamine (LDN) is a selective ligand for Gal-3. The terminal LDN motif in the oligosaccharide chain is an efficient way to increase selectivity for Gal-3. Even in glycopolymer **G7** with the lowest LDN-LN2 content, the affinity to Gal-3 was quite high, over 900-fold higher than to Gal-1. Also, for glycopolymer **G8** with the low LDN-LN2 content, the selectivity for Gal-3 was again twice as high as in **G7** due to the increased binding affinity for Gal-3. Compared with other previously published glycoconjugates suitable for in vivo applications, such as polymer scaffolds of HPMA-based copolymers or polyoxazolines carrying glycomimetic ligands of galectins,^38^,^53^ or with other disaccharide-presenting carriers such as glycodendrimers and glycocalixarenes,^54^,^55^ the present compounds show a significantly higher affinity (by at least one order of magnitude), and, in particular, a much more pronounced selectivity to Gal-3 (by at least 2–3 orders of magnitude or more). Moreover, the hydrophilic nature of oligosaccharide ligands prevents problems with limited solubility and uncontrolled micellization, clustering and/or aggregation of the glycopolymer sample, which are undesirable properties also in view of a potential in vivo application. Though protein scaffolds such as human serum albumin seem to be slightly more efficient in terms of affinities^17^,^22^ for the multivalent presentation of galectin ligands, these protein scaffolds lack the favorable properties of glycopolymers including intracellular permeability, which apparently influences a vast number of galectin-associated processes by interaction with intracellular galectin. Hence, we consider the present glycopolymer concept as an optimal choice for further progress towards more advanced studies, including in vivo applications.

### Inhibition of Gal-3-Induced Apoptosis of Macrophages

**Figure 2:**
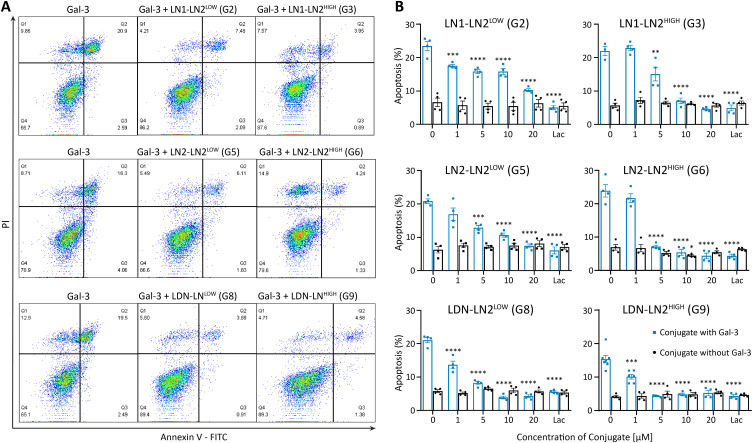
Inhibition of Gal-3-induced apoptosis of macrophages by glycopolymers. Addition of recombinant Gal-3 induces apoptosis to ca 15–25% of macrophages. This process is dose-dependently prevented by the addition of glycopolymers that capture Gal-3 and do not allow it to bind to macrophages. (**A**) Representative flow cytometry scatter plots and gating strategy of J774A.1 macrophages treated with 10 µM Gal-3 only or in combination with 20 µM glycopolymer **G2, G3** (upper row), **G5, G6** (middle row), or **G8, G9** (bottom row), respectively. Early apoptotic and late apoptotic macrophage cells are present in the Q3 or Q2 quadrant, respectively. Decrease in percentage of cells especially in Q2 quadrant in the absence (Gal-3 column) and in the presence of all glycopolymers (Gal-3 + **G2** **– G9** column) is clearly visible. (**B**) Flow cytometry analysis of apoptosis (sum of early and late apoptosis, ie percentage of apoptotic cells in Q3 and Q2 quadrant) of J774A.1 macrophages treated with 10 µM Gal-3 only or in combination with 0–20 µM glycopolymers **G2, G3** (upper row), **G5, G6** (middle row), or **G8, G9** (bottom row), respectively. Bar graphs indicate the mean ± SEM (n = 2, statistical analysis by one-way ANOVA with Dunnett’s post hoc test; *P < 0.05, **P < 0.01, ***P < 0.001, ****P < 0.0001 *versus* control).

Tumor-associated macrophages (TAMs) are defined as M1 or M2 subtypes, which differ in their roles in carcinogenesis.^56^ Phenotypically M2 macrophages, which are usually more abundant inside tumors and seem to be mainly responsible for the spread of cancer,^57^ are chemoattracted into the tumor mass by the Gal-3 gradient.^11^ Therefore, we aimed to investigate the inhibitory potential of prepared glycopolymers towards Gal-3 in the context of monocytes and macrophages. For this purpose, we used J774A.1 macrophage cell line, which exhibits a characteristic of the intermediate monocyte-macrophage stage of development, ie, a rather more undifferentiated and unpolarized (M0 state),^58^,^59^ and we studied if Gal-3 induced apoptosis in these cells. The 10 µM concentration of Gal-3 induced average apoptosis of J774A.1 cells up to 20.7 ± 0.8% (n = 12). We assessed the performance of glycopolymers with low (≈ 4–5 mol. %) and high (≈ 8–10 mol. %) content of the tetrasaccharide motif of **LN1-LN2** (**G2, G3**), **LN2-LN2** (**G5, G6**), and **LDN-LN2** (**G8, G9**) in inhibiting Gal-3-induced apoptosis of undifferentiated J774A.1 macrophages (Figure 2). We found that different tetrasaccharide motifs led to significant differences in the inhibitory efficiency of the glycopolymers. All the glycopolymers, including also the polymeric carrier itself, were non-toxic (Figure S6). The glycopolymers with a high tetrasaccharide content (≈ 10 mol. %) mostly inhibited apoptosis in a lower concentration than those with a low tetrasaccharide content. Whereas **LN2-LN2**- and **LDN-LN2-**decorated glycopolymers **G6** and **G9**, respectively, with a high tetrasaccharide content completely inhibited apoptosis induced by 10 µM Gal-3 in a concentration of 5 µM (Table 2), **LN1-LN2-**decorated glycopolymer **G3** reached complete inhibition of Gal-3 in a concentration of 10 µM. The differences in the inhibitory potency were much more pronounced in glycopolymers with a low content of tetrasaccharide moieties. **LN1-LN2**-decorated glycopolymer **G2** was not able to evoke complete inhibition of apoptosis even in the highest used 20 µM concentration. In contrast, glycopolymer **G5** decorated with the **LN2-LN2** motif completely inhibited Gal-3 in this concentration. The best-performing low-content tetrasaccharide glycopolymer was **G8** (with **LDN-LN2** motif), which fully inhibited the apoptosis of macrophages in a concentration of 5 µM. Surprisingly, the differences in the inhibitory capabilities between low- and high-content tetrasaccharide glycopolymers **G8** and **G9** were negligible, and quite small differences were also seen between **G5** and **G6**. This may be caused by a certain saturation of the observed anti-apoptotic effect – apparently, the glycan number in the low-content-tetrasaccharide glycopolymers is already quite sufficient to protect macrophages against apoptosis and additional increase in the glycan number does not bring any further enhancement. A more distinct difference lies undebatably in the choice of a particular glycan (cf. **LN1-LN2** vs **LN2-LN2** and, in particular, the most efficient motif **LDN-LN2**). In this sense, the anti-apoptotic effect is more robust than a simple affinity measurement by ELISA, which shows more subtle differences. In sum, the glycopolymers could be ranked in the order of potency as follows: **LDN-LN2** > **LN2-LN2** > **LN1-LN2**.

**Table 2:** IC_50_ Values Derived from Dose-Response Curves of Inhibition of Gal-3-Induced Apoptosis by Glycopolymers

Ligand Motif	LN1-LN2		LN2-LN2		LDN-LN2	
Glycopolymer	G2	G3	G5	G6	G8	G9
Content [mol. %]	4.0 (low)	7.8 (high)	4.4 (low)	7.9 (high)	4.9 (low)	9.7 (high)
IC_50_^a^ [µM]	7.9 ± 0.7	4.7 ± 1.1	3.5 ± 1.7	1.6 ± 0.2	1.1 ± 0.3	1.1 ± 0.1

**Notes**: ^a^ IC_50_ values were determined using nonlinear regression analysis.

These results demonstrate that Gal-3 is capable of directly inducing apoptosis, not only in T cells as already reported,^16^,^60^,^61^ but also in the naïve unpolarized M0 macrophages (J774A.1 cells). Although the precise molecular pathway of the Gal-3-induced apoptosis has not been fully disclosed yet, especially in macrophages, a possible mechanism might be through the direct interaction of Gal-3 with surface cell glycoproteins CD29 and CD7 in the CD29/27 complex as described for the extracellular Gal-3.^62^ This induces classical intracellular apoptotic pathway through mitochondria, release of cytochrome c and activation of caspases. Sustained retention of a high proportion of live, non-apoptotic M0 and M1 macrophages in the tumor mass is highly desirable, particularly due to the direct antitumorigenic activities of M0 macrophages^63^ and the indirect ability of M1 macrophages to induce tumor cell lysis, probably by supporting the antitumor Th1 response and the migration of cytotoxic T lymphocytes into the tumor site.^64^ Therefore, the glycopolymers presented here may be very helpful in suppressing the Gal-3 gradient generated by the tumor mass, which chemoattracts unwanted M2-like TAMs;^11^ on the one hand they may enable direct inhibition of Gal-3-induced apoptosis of macrophages, possibly leading to an increase in the proportion of macrophages in the tumor mass.

### Inhibition of Gal-3-Induced Capture of IFNγ

**Figure 3:**
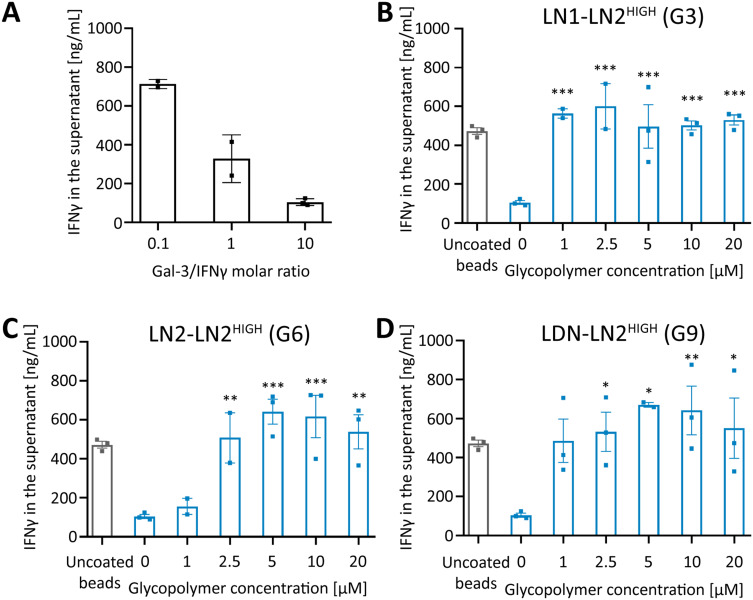
Competitive inhibition of Gal-3-mediated IFNγ capture by glycopolymers. (**A)** Dose-response decrease in IFNγ concentration after incubation with Gal-3-coated magnetic beads. (**B–D**) Concentration of residual IFNγ after incubation with Gal-3-coated beads (Gal-3/IFNγ molar ratio 1/10) with glycopolymer **G3** (**B), G6** (**C**) or **G9** (**D**) as determined by ELISA. Graphs represent means ± SEM (n = 3; statistical analysis by one-way ANOVA with Dunnett’s post hoc test; *P < 0.05, **P < 0.01, ***P < 0.001 *versus* control). Black columns - uncoated magnetic beads, blue columns – Gal-3-coated magnetic beads.

Tumor-supportive M2 monocytes presented in TME differentiate from monocytes recruited by TME.^65^ However, monocytes can differentiate into anti-tumor M1 pro-inflammatory macrophages upon the influence of IFNγ produced by CD8+ T cells, natural killer (NK) cells and T helper (Th) cells.^65^ Since Gal-3 can bind glycosylated proteins, we investigated the ability of the prepared glycopolymers to competitively inhibit Gal-3-induced capture of glycosylated human IFNγ, which means that IFNγ is further available for monocytes to differentiate into M1 subtype. Adopting the previously published protocol,^12^ we immobilized the monobiotinylated Gal-3 construct on streptavidin magnetic beads through biotin-streptavidin interaction. We first verified whether immobilized Gal-3 could capture IFNγ from the solution. We found that the increasing amount of Gal-3-coated magnetic beads in the solution caused a dose–response decrease of IFNγ concentration in the solution as detected by the ELISA kit (Figure 3A). When glycopolymers **G3, G6**, or **G9** with a high ligand content were added to the solution containing IFNγ and Gal-3-magnetic beads, glycopolymers competed with IFNγ for Gal-3 and thus disabled the capture of IFNγ by Gal-3 (Figure 3B–D). The concentration range of 2.5–20 µM of all tested glycopolymers completely inhibited the Gal-3-mediated IFNγ capture. In the concentration of 1 µM, the differences between glycopolymers carrying different tetrasaccharide ligands were more pronounced. Glycopolymers **G3** and **G9** with ligand motifs **LDN-LN2** and **LN1-LN2** fully inhibited Gal-3-IFNγ interaction already in a concentration of 1 µM, whereas a higher concentration was required for glycopolymer **G6** with the **LN2-LN2** motif.

A limitation of our in vitro study is the use of commercially available immune cells isolated from the patients with tumors (ie cell lines), which just represent a simplified model of the complex situation in vivo. The possible challenges in the application of glycopolymers in vivo or clinically might be their accumulation in other organs, such as the liver or kidney, which might cause off-target effects. Additional work will be needed to examine all the mechanisms of action in vivo and establish possible side effects and the connection of glycopolymers to the immune resolution of tumors. The presence of glycopolymers inside the tumor mass needs to be sufficient to weaken the TME and enable the infiltration of immune cells. It has been shown previously that HPMA-based polymer carriers accumulate in tumor mass through the EPR effect.^28^ Therefore, the present highly efficient glycopolymers may inhibit the Gal-3-induced apoptosis of tumor-contained macrophages and restore the biological functions of Gal-3-captured IFNγ in the tumors. This approach may open the pathway to use anti-PD-1 blockage (or another immune checkpoint inhibitor therapy) for the treatment of solid tumors to increase the effectiveness of the state-of-the-art immune antitumor therapies. For example, it has been shown that the inhibition of Gal-3 supports the infiltration of T cells and increases the sensitivity of immune checkpoint inhibitor therapy (anti-PD-L1).^66^ Similarly, Gal-3 is able to reduce the binding of anti-PD-1 (pembrolizumab) and anti-PD-L1 (atezolizumab). The utilization of a potent inhibitor restores the binding abilities of anti-PD-1 and anti-PD-L1.^15^ Glycopolymers, which are potent Gal-3 inhibitors, should have the same effect and cooperate in synergy with the currently used immune checkpoint inhibitor therapies or in combination with immunotherapy using antibody-drug conjugates.

## Conclusions

We have designed, synthesized, and evaluated a set of biocompatible water-soluble HPMA-based glycopolymers decorated with varying content of three types of poly-LacNAc-derived tetrasaccharide moieties, β3Gal-capped **LN1-LN2**, β4Gal-capped **LN2-LN2**, and β4GalNAc-capped **LDN-LN2**. We demonstrated a high binding affinity and selectivity of the prepared glycopolymers for the immunomodulatory protein Gal-3, which increased in the order **LN1-LN2 ˂ LN2-LN2** ˂ **LDN-LN2**. The best glycopolymer achieved an IC_50_ of 23 nM and an over 1800-fold selectivity for Gal-3 over Gal-1. We further demonstrate that the glycopolymers completely inhibited Gal-3-induced apoptosis of tumor-contained macrophages and very efficiently blocked Gal-3-mediated capture of IFNγ, which is essential for the differentiation of monocytes into pro-inflammatory (anti-tumorigenic) monocytes and/or macrophages. Our data suggest that the prepared glycopolymers play a distinct role in modulating the immunosuppressive TME to a favorable pro-inflammatory state, in which M0/M1 monocytes and macrophages are protected against Gal-3-induced apoptosis and IFNγ is available since its capture by Gal-3 is blocked. IFNγ-driven activation of M1 macrophages induces the ability of present antigens, activates CD8^+^ T cell proliferation, stimulates their effector functions, and brings CD8^+^ T and NK cells into the tumor. Consequently, the presence of macrophages M1 inside the tumor might increase survival prognosis and improve response to immune checkpoint inhibitors immunotherapy of patients with cancer. Developed glycopolymers thus represent a novel class of nanotherapeutics, which can serve as supportive nanosystems in the complex treatment of advanced cancer diseases. Therefore, our future studies will be aimed at the application of glycopolymers in vivo, especially their biodistribution, biocompatibility, and synergic antitumorigenic effect in combination with antibody-drug-conjugate immunotherapies.
